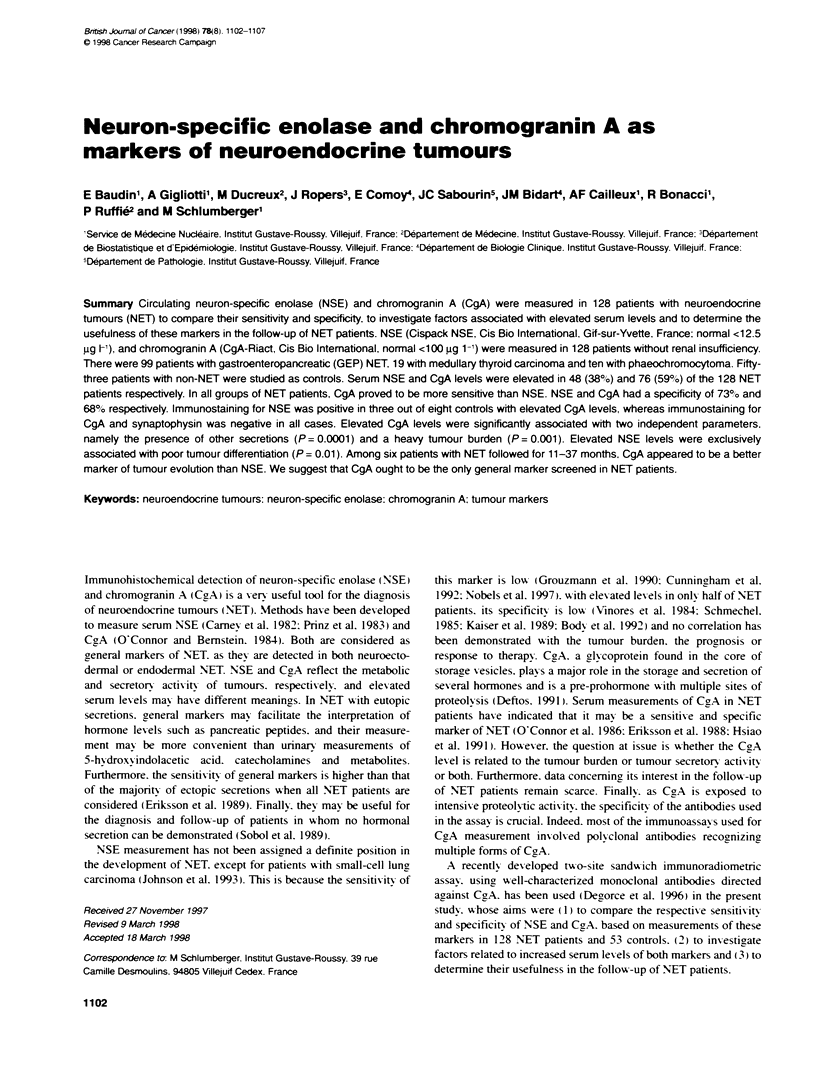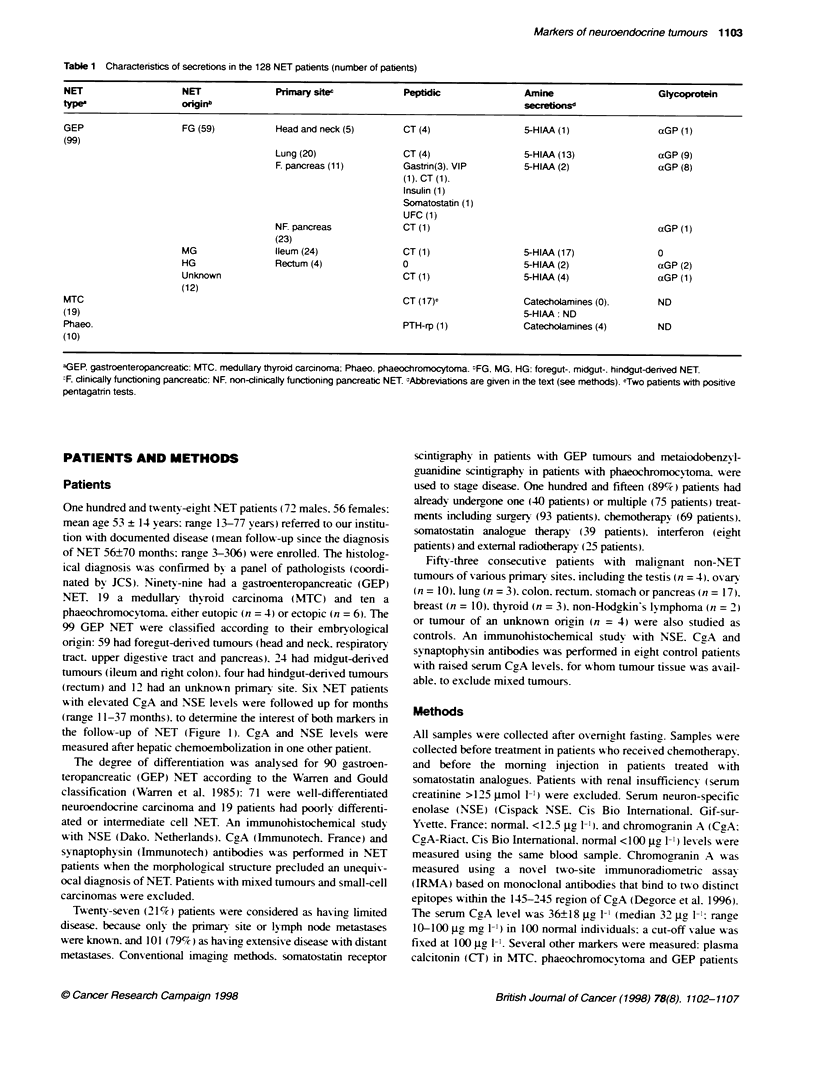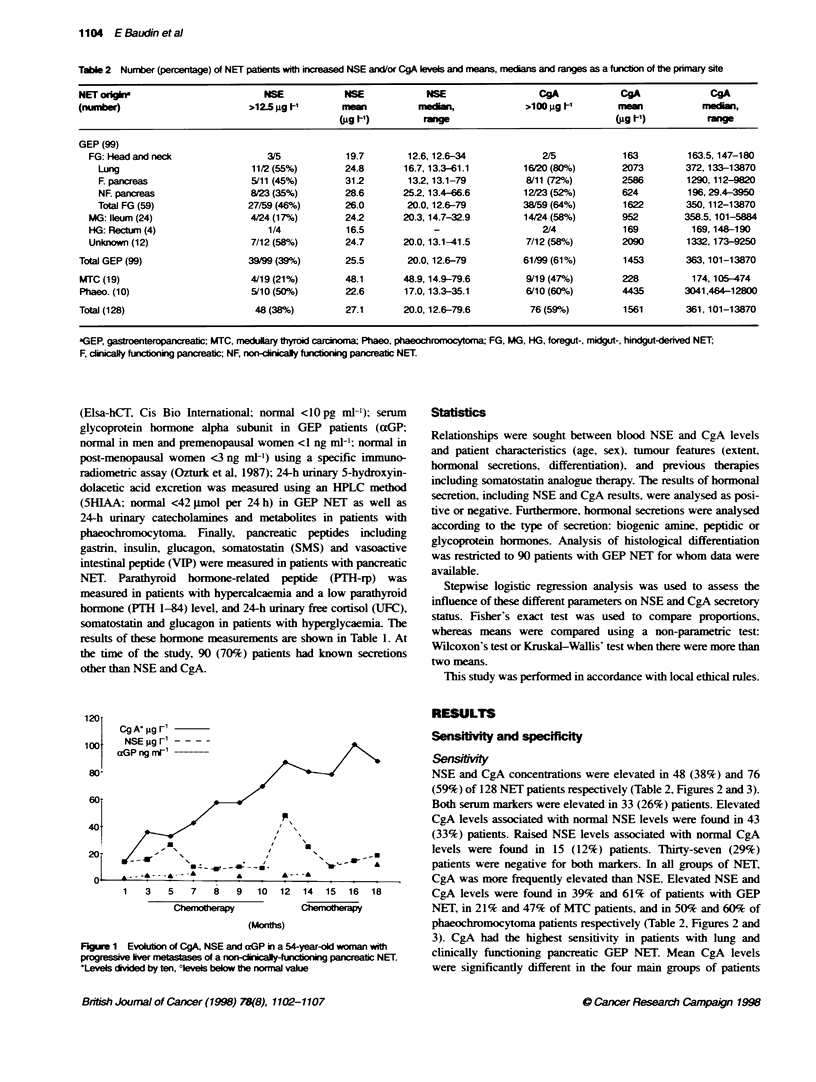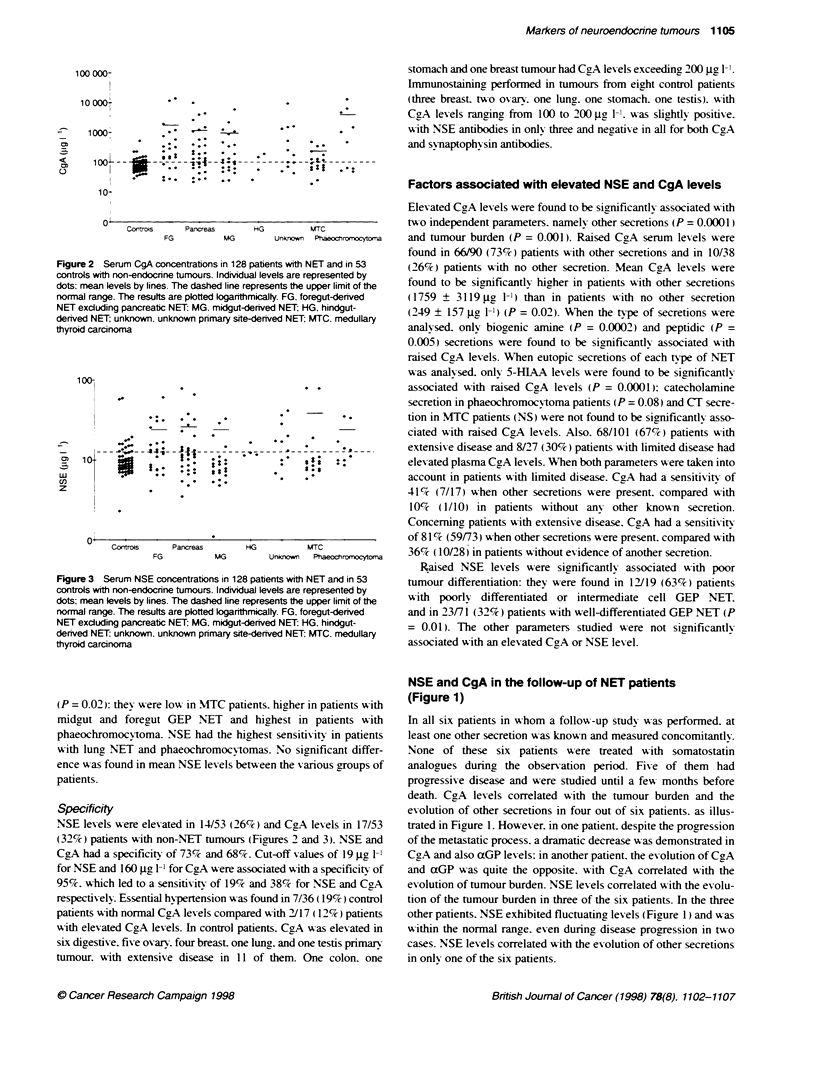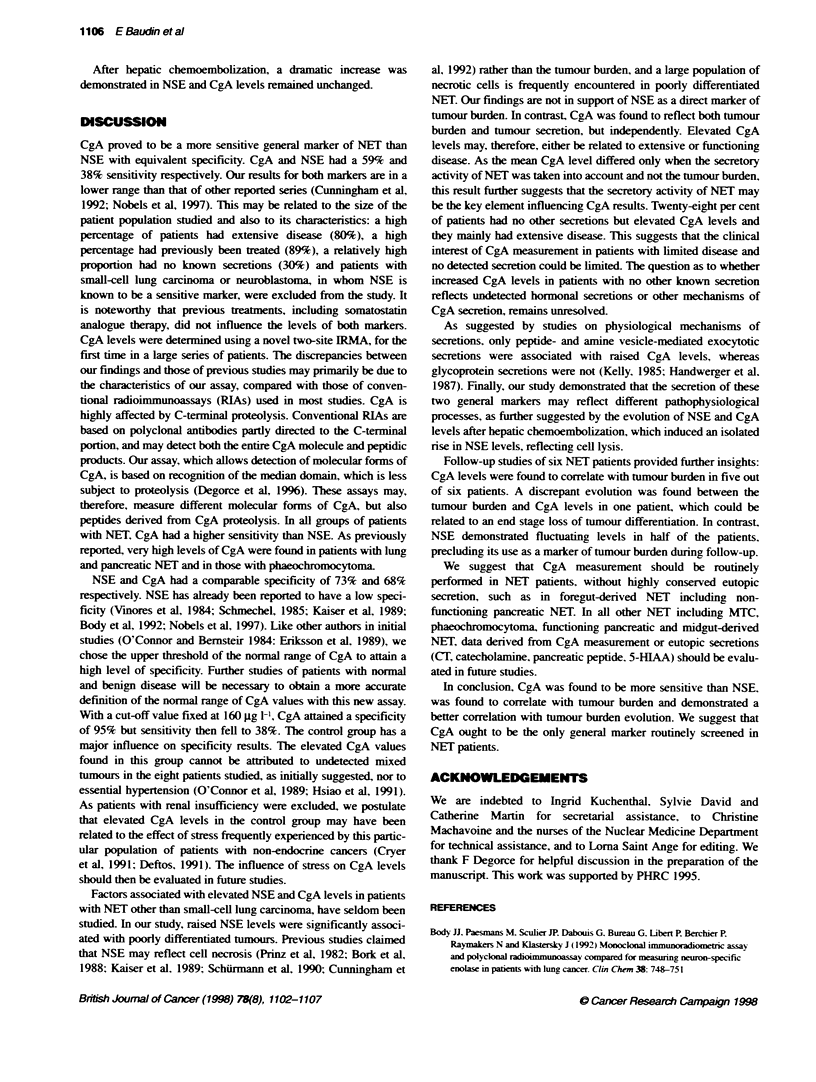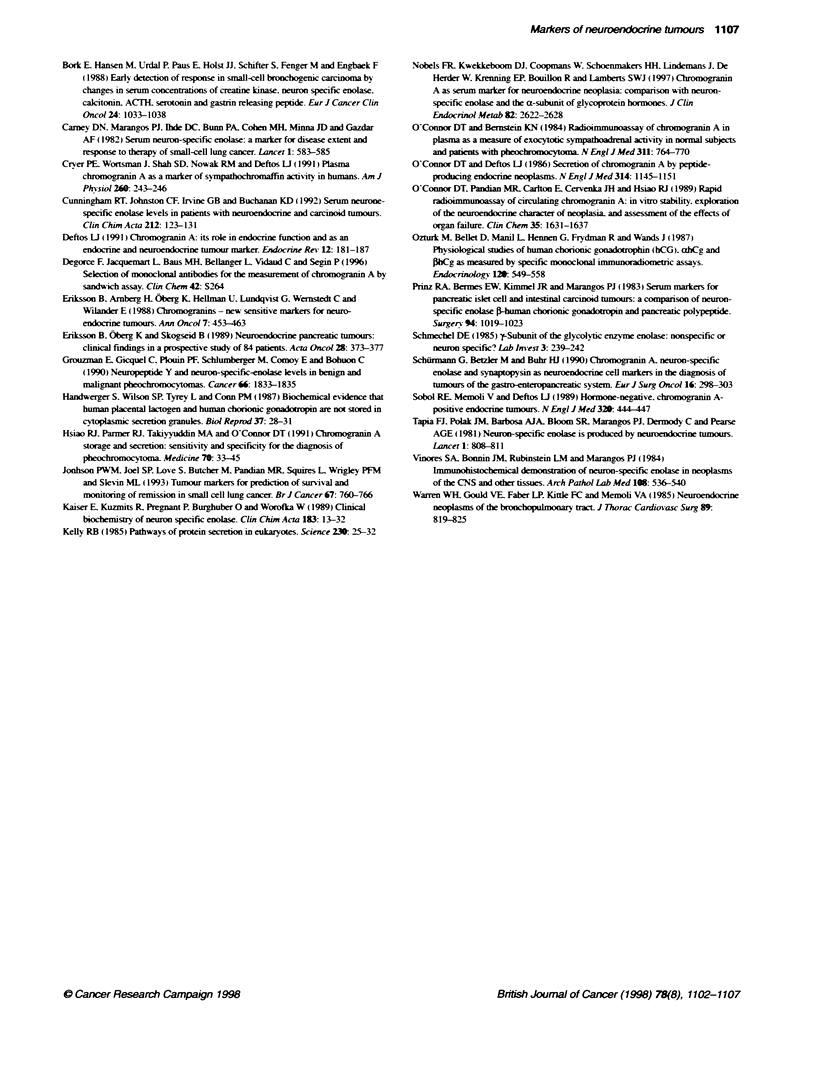# Neuron-specific enolase and chromogranin A as markers of neuroendocrine tumours.

**DOI:** 10.1038/bjc.1998.635

**Published:** 1998-10

**Authors:** E. Baudin, A. Gigliotti, M. Ducreux, J. Ropers, E. Comoy, J. C. Sabourin, J. M. Bidart, A. F. Cailleux, R. Bonacci, P. Ruffié, M. Schlumberger

**Affiliations:** Service de Médecine Nucléaire, Institut Gustave-Roussy, Villejuif, France.

## Abstract

Circulating neuron-specific enolase (NSE) and chromogranin A (CgA) were measured in 128 patients with neuroendocrine tumours (NET) to compare their sensitivity and specificity, to investigate factors associated with elevated serum levels and to determine the usefulness of these markers in the follow-up of NET patients. NSE (Cispack NSE, Cis Bio International, Gif-sur-Yvette, France; normal <12.5 microg l(-1)), and chromogranin A (CgA-Riact, Cis Bio International, normal <100 microg l(-1)) were measured in 128 patients without renal insufficiency. There were 99 patients with gastroenteropancreatic (GEP) NET, 19 with medullary thyroid carcinoma and ten with phaeochromocytoma. Fifty-three patients with non-NET were studied as controls. Serum NSE and CgA levels were elevated in 48 (38%) and 76 (59%) of the 128 NET patients respectively. In all groups of NET patients, CgA proved to be more sensitive than NSE. NSE and CgA had a specificity of 73% and 68% respectively. Immunostaining for NSE was positive in three out of eight controls with elevated CgA levels, whereas immunostaining for CgA and synaptophysin was negative in all cases. Elevated CgA levels were significantly associated with two independent parameters, namely the presence of other secretions (P = 0.0001) and a heavy tumour burden (P = 0.001). Elevated NSE levels were exclusively associated with poor tumour differentiation (P = 0.01). Among six patients with NET followed for 11-37 months, CgA appeared to be a better marker of tumour evolution than NSE. We suggest that CgA ought to be the only general marker screened in NET patients.


British Journal of Cancer (1998) 78(8), 1102–1107
© 1998 Cancer Research Campaign

### Neuron-specific enolase and chromogranin A as markers of neuroendocrine tumours

**E Baudin[1], A Gigliotti[1], M Ducreux[2], J Ropers[3], E Comoy[4], JC Sabourin[5], JM Bidart[4], AF Cailleux[1], R Bonacci[1], P Ruffié[2] and M Schlumberger[1]**

[1]Service de Médecine Nucléaire, Institut Gustave-Roussy, Villejuif, France; [2]Département de Médecine, Institut Gustave-Roussy, Villejuif, France; [3]Département de Biostatistique et d'Epidémiologie, Institut Gustave-Roussy, Villejuif, France; [4]Département de Biologie Clinique, Institut Gustave-Roussy, Villejuif, France; [5]Département de Pathologie, Institut Gustave-Roussy, Villejuif, France

**Summary** Circulating neuron-specific enolase (NSE) and chromogranin A (CgA) were measured in 128 patients with neuroendocrine tumours (NET) to compare their sensitivity and specificity, to investigate factors associated with elevated serum levels and to determine the usefulness of these markers in the follow-up of NET patients. NSE (Cispack NSE, Cis Bio International, Gif-sur-Yvette, France; normal <12.5 μg $l^{-1}$), and chromogranin A (CgA-Riact, Cis Bio International, normal <100 μg $l^{-1}$) were measured in 128 patients without renal insufficiency. There were 99 patients with gastroenteropancreatic (GEP) NET, 19 with medullary thyroid carcinoma and ten with phaeochromocytoma. Fifty-three patients with non-NET were studied as controls. Serum NSE and CgA levels were elevated in 48 (38%) and 76 (59%) of the 128 NET patients respectively. In all groups of NET patients, CgA proved to be more sensitive than NSE. NSE and CgA had a specificity of 73% and 68% respectively. Immunostaining for NSE was positive in three out of eight controls with elevated CgA levels, whereas immunostaining for CgA and synaptophysin was negative in all cases. Elevated CgA levels were significantly associated with two independent parameters, namely the presence of other secretions ($P = 0.0001$) and a heavy tumour burden ($P = 0.001$). Elevated NSE levels were exclusively associated with poor tumour differentiation ($P = 0.01$). Among six patients with NET followed for 11–37 months, CgA appeared to be a better marker of tumour evolution than NSE. We suggest that CgA ought to be the only general marker screened in NET patients.

**Keywords:** neuroendocrine tumours; neuron-specific enolase; chromogranin A; tumour markers

Immunohistochemical detection of neuron-specific enolase (NSE) and chromogranin A (CgA) is a very useful tool for the diagnosis of neuroendocrine tumours (NET). Methods have been developed to measure serum NSE (Carney et al. 1982; Prinz et al. 1983) and CgA (O'Connor and Bernstein, 1984). Both are considered as general markers of NET, as they are detected in both neuroectodermal or endodermal NET. NSE and CgA reflect the metabolic and secretory activity of tumours, respectively, and elevated serum levels may have different meanings. In NET with eutopic secretions, general markers may facilitate the interpretation of hormone levels such as pancreatic peptides, and their measurement may be more convenient than urinary measurements of 5-hydroxyindolacetic acid, catecholamines and metabolites. Furthermore, the sensitivity of general markers is higher than that of the majority of ectopic secretions when all NET patients are considered (Eriksson et al. 1989). Finally, they may be useful for the diagnosis and follow-up of patients in whom no hormonal secretion can be demonstrated (Sobol et al. 1989).

NSE measurement has not been assigned a definite position in the development of NET, except for patients with small-cell lung carcinoma (Johnson et al. 1993). This is because the sensitivity of this marker is low (Grouzmann et al. 1990; Cunningham et al. 1992; Nobels et al. 1997), with elevated levels in only half of NET patients, its specificity is low (Vinores et al. 1984; Schmechel, 1985; Kaiser et al. 1989; Body et al. 1992) and no correlation has been demonstrated with the tumour burden, the prognosis or response to therapy. CgA, a glycoprotein found in the core of storage vesicles, plays a major role in the storage and secretion of several hormones and is a pre-prohormone with multiple sites of proteolysis (Deftos, 1991). Serum measurements of CgA in NET patients have indicated that it may be a sensitive and specific marker of NET (O'Connor et al. 1986; Eriksson et al. 1988; Hsiao et al. 1991). However, the question at issue is whether the CgA level is related to the tumour burden or tumour secretory activity or both. Furthermore, data concerning its interest in the follow-up of NET patients remain scarce. Finally, as CgA is exposed to intensive proteolytic activity, the specificity of the antibodies used in the assay is crucial. Indeed, most of the immunoassays used for CgA measurement involved polyclonal antibodies recognizing multiple forms of CgA.

A recently developed two-site sandwich immunoradiometric assay, using well-characterized monoclonal antibodies directed against CgA, has been used (Degorce et al. 1996) in the present study, whose aims were (1) to compare the respective sensitivity and specificity of NSE and CgA, based on measurements of these markers in 128 NET patients and 53 controls, (2) to investigate factors related to increased serum levels of both markers and (3) to determine their usefulness in the follow-up of NET patients.

*Received 27 November 1997*
*Revised 9 March 1998*
*Accepted 18 March 1998*

*Correspondence to:* M Schlumberger, Institut Gustave-Roussy, 39 rue Camille Desmoulins, 94805 Villejuif Cedex, France

**Table 1** Characteristics of secretions in the 128 NET patients (number of patients)

| NET type[a] | NET origin[b] | Primary site[c] | Peptidic | Amine secretions[d] | Glycoprotein |
|---|---|---|---|---|---|
| GEP (99) | FG (59) | Head and neck (5) | CT (4) | 5-HIAA (1) | αGP (1) |
| | | Lung (20) | CT (4) | 5-HIAA (13) | αGP (9) |
| | | F. pancreas (11) | Gastrin(3). VIP (1). CT (1). Insulin (1) Somatostatin (1) UFC (1) | 5-HIAA (2) | αGP (8) |
| | | NF. pancreas (23) | CT (1) | | αGP (1) |
| | MG | Ileum (24) | CT (1) | 5-HIAA (17) | 0 |
| | HG | Rectum (4) | 0 | 5-HIAA (2) | αGP (2) |
| | Unknown (12) | | CT (1) | 5-HIAA (4) | αGP (1) |
| MTC (19) | | | CT (17)[e] | Catecholamines (0). 5-HIAA : ND | ND |
| Phaeo. (10) | | | PTH-rp (1) | Catecholamines (4) | ND |

[a]GEP. gastroenteropancreatic: MTC. medullary thyroid carcinoma: Phaeo. phaeochromocytoma. [b]FG. MG. HG: foregut-. midgut-. hindgut-derived NET. [c]F. clinically functioning pancreatic: NF. non-clinically functioning pancreatic NET. [d]Abbreviations are given in the text (see methods). [e]Two patients with positive pentagatrin tests.

### PATIENTS AND METHODS

### Patients

One hundred and twenty-eight NET patients (72 males. 56 females: mean age 53 ± 14 years: range 13–77 years) referred to our institution with documented disease (mean follow-up since the diagnosis of NET 56±70 months: range 3–306) were enrolled. The histological diagnosis was confirmed by a panel of pathologists (coordinated by JCS). Ninety-nine had a gastroenteropancreatic (GEP) NET. 19 a medullary thyroid carcinoma (MTC) and ten a phaeochromocytoma. either eutopic ($n$ = 4) or ectopic ($n$ = 6). The 99 GEP NET were classified according to their embryological origin: 59 had foregut-derived tumours (head and neck. respiratory tract. upper digestive tract and pancreas). 24 had midgut-derived tumours (ileum and right colon). four had hindgut-derived tumours (rectum) and 12 had an unknown primary site. Six NET patients with elevated CgA and NSE levels were followed up for months (range 11–37 months). to determine the interest of both markers in the follow-up of NET (Figure 1). CgA and NSE levels were measured after hepatic chemoembolization in one other patient.

The degree of differentiation was analysed for 90 gastroenteropancreatic (GEP) NET according to the Warren and Gould classification (Warren et al. 1985): 71 were well-differentiated neuroendocrine carcinoma and 19 patients had poorly differentiated or intermediate cell NET. An immunohistochemical study with NSE (Dako. Netherlands). CgA (Immunotech. France) and synaptophysin (Immunotech) antibodies was performed in NET patients when the morphological structure precluded an unequivocal diagnosis of NET. Patients with mixed tumours and small-cell carcinomas were excluded.

Twenty-seven (21%) patients were considered as having limited disease. because only the primary site or lymph node metastases were known. and 101 (79%) as having extensive disease with distant metastases. Conventional imaging methods. somatostatin receptor scintigraphy in patients with GEP tumours and metaiodobenzylguanidine scintigraphy in patients with phaeochromocytoma. were used to stage disease. One hundred and fifteen (89%) patients had already undergone one (40 patients) or multiple (75 patients) treatments including surgery (93 patients). chemotherapy (69 patients). somatostatin analogue therapy (39 patients). interferon (eight patients) and external radiotherapy (25 patients).

Fifty-three consecutive patients with malignant non-NET tumours of various primary sites. including the testis ($n$ = 4). ovary ($n$ = 10). lung ($n$ = 3). colon. rectum. stomach or pancreas ($n$ = 17). breast ($n$ = 10). thyroid ($n$ = 3). non-Hodgkin's lymphoma ($n$ = 2) or tumour of an unknown origin ($n$ = 4) were also studied as controls. An immunohistochemical study with NSE. CgA and synaptophysin antibodies was performed in eight control patients with raised serum CgA levels. for whom tumour tissue was available. to exclude mixed tumours.

### Methods

All samples were collected after overnight fasting. Samples were collected before treatment in patients who received chemotherapy. and before the morning injection in patients treated with somatostatin analogues. Patients with renal insufficiency (serum creatinine >125 μmol $l^{-1}$) were excluded. Serum neuron-specific enolase (NSE) (Cispack NSE. Cis Bio International. Gif-sur-Yvette. France: normal. <12.5 μg $l^{-1}$). and chromogranin A (CgA: CgA-Riact. Cis Bio International. normal <100 μg $l^{-1}$) levels were measured using the same blood sample. Chromogranin A was measured using a novel two-site immunoradiometric assay (IRMA) based on monoclonal antibodies that bind to two distinct epitopes within the 145–245 region of CgA (Degorce et al. 1996). The serum CgA level was 36±18 μg $l^{-1}$ (median 32 μg $l^{-1}$: range 10–100 μg mg $l^{-1}$) in 100 normal individuals: a cut-off value was fixed at 100 μg $l^{-1}$. Several other markers were measured: plasma calcitonin (CT) in MTC. phaeochromocytoma and GEP patients

© Cancer Research Campaign 1998

Table 2 Number (percentage) of NET patients with increased NSE and/or CgA levels and means, medians and ranges as a function of the primary site

| NET origin* (number) | NSE >12.5 µg l⁻¹ | NSE mean (µg l⁻¹) | NSE median, range | CgA >100 µg l⁻¹ | CgA mean (µg l⁻¹) | CgA median, range |
|---|---|---|---|---|---|---|
| GEP (99) | | | | | | |
| FG: Head and neck | 3/5 | 19.7 | 12.6, 12.6–34 | 2/5 | 163 | 163.5, 147–180 |
| Lung | 11/2 (55%) | 24.8 | 16.7, 13.3–61.1 | 16/20 (80%) | 2073 | 372, 133–13870 |
| F. pancreas | 5/11 (45%) | 31.2 | 13.2, 13.1–79 | 8/11 (72%) | 2586 | 1290, 112–9820 |
| NF. pancreas | 8/23 (35%) | 28.6 | 25.2, 13.4–66.6 | 12/23 (52%) | 624 | 196, 29.4–3950 |
| Total FG (59) | 27/59 (46%) | 26.0 | 20.0, 12.6–79 | 38/59 (64%) | 1622 | 350, 112–13870 |
| MG: Ileum (24) | 4/24 (17%) | 24.2 | 20.3, 14.7–32.9 | 14/24 (58%) | 952 | 358.5, 101–5884 |
| HG: Rectum (4) | 1/4 | 16.5 | – | 2/4 | 169 | 169, 148–190 |
| Unknown (12) | 7/12 (58%) | 24.7 | 20.0, 13.1–41.5 | 7/12 (58%) | 2090 | 1332, 173–9250 |
| Total GEP (99) | 39/99 (39%) | 25.5 | 20.0, 12.6–79 | 61/99 (61%) | 1453 | 363, 101–13870 |
| MTC (19) | 4/19 (21%) | 48.1 | 48.9, 14.9–79.6 | 9/19 (47%) | 228 | 174, 105–474 |
| Phaeo. (10) | 5/10 (50%) | 22.6 | 17.0, 13.3–35.1 | 6/10 (60%) | 4435 | 3041,464–12800 |
| Total (128) | 48 (38%) | 27.1 | 20.0, 12.6–79.6 | 76 (59%) | 1561 | 361, 101–13870 |

*GEP, gastroenteropancreatic; MTC, medullary thyroid carcinoma; Phaeo, phaeochromocytoma; FG, MG, HG, foregut-, midgut-, hindgut-derived NET; F, clinically functioning pancreatic; NF, non-clinically functioning pancreatic NET.

(Elsa-hCT, Cis Bio International; normal <10 pg ml⁻¹); serum glycoprotein hormone alpha subunit in GEP patients (αGP; normal in men and premenopausal women <1 ng ml⁻¹; normal in post-menopausal women <3 ng ml⁻¹) using a specific immunoradiometric assay (Ozturk et al, 1987); 24-h urinary 5-hydroxyindolacetic acid excretion was measured using an HPLC method (5HIAA; normal <42 µmol per 24 h) in GEP NET as well as 24-h urinary catecholamines and metabolites in patients with phaeochromocytoma. Finally, pancreatic peptides including gastrin, insulin, glucagon, somatostatin (SMS) and vasoactive intestinal peptide (VIP) were measured in patients with pancreatic NET. Parathyroid hormone-related peptide (PTH-rp) was measured in patients with hypercalcaemia and a low parathyroid hormone (PTH 1–84) level, and 24-h urinary free cortisol (UFC), somatostatin and glucagon in patients with hyperglycaemia. The results of these hormone measurements are shown in Table 1. At the time of the study, 90 (70%) patients had known secretions other than NSE and CgA.

Figure 1 Evolution of CgA, NSE and αGP in a 54-year-old woman with progressive liver metastases of a non-clinically-functioning pancreatic NET. *Levels divided by ten, °levels below the normal value

### Statistics

Relationships were sought between blood NSE and CgA levels and patient characteristics (age, sex), tumour features (extent, hormonal secretions, differentiation), and previous therapies including somatostatin analogue therapy. The results of hormonal secretion, including NSE and CgA results, were analysed as positive or negative. Furthermore, hormonal secretions were analysed according to the type of secretion: biogenic amine, peptidic or glycoprotein hormones. Analysis of histological differentiation was restricted to 90 patients with GEP NET for whom data were available.

Stepwise logistic regression analysis was used to assess the influence of these different parameters on NSE and CgA secretory status. Fisher's exact test was used to compare proportions, whereas means were compared using a non-parametric test: Wilcoxon's test or Kruskal–Wallis' test when there were more than two means.

This study was performed in accordance with local ethical rules.

### RESULTS

### Sensitivity and specificity

### *Sensitivity*

NSE and CgA concentrations were elevated in 48 (38%) and 76 (59%) of 128 NET patients respectively (Table 2, Figures 2 and 3). Both serum markers were elevated in 33 (26%) patients. Elevated CgA levels associated with normal NSE levels were found in 43 (33%) patients. Raised NSE levels associated with normal CgA levels were found in 15 (12%) patients. Thirty-seven (29%) patients were negative for both markers. In all groups of NET, CgA was more frequently elevated than NSE. Elevated NSE and CgA levels were found in 39% and 61% of patients with GEP NET, in 21% and 47% of MTC patients, and in 50% and 60% of phaeochromocytoma patients respectively (Table 2, Figures 2 and 3). CgA had the highest sensitivity in patients with lung and clinically functioning pancreatic GEP NET. Mean CgA levels were significantly different in the four main groups of patients

**Figure 2** Serum CgA concentrations in 128 patients with NET and in 53 controls with non-endocrine tumours. Individual levels are represented by dots; mean levels by lines. The dashed line represents the upper limit of the normal range. The results are plotted logarithmically. FG, foregut-derived NET excluding pancreatic NET; MG, midgut-derived NET; HG, hindgut-derived NET; unknown, unknown primary site-derived NET; MTC, medullary thyroid carcinoma

**Figure 3** Serum NSE concentrations in 128 patients with NET and in 53 controls with non-endocrine tumours. Individual levels are represented by dots; mean levels by lines. The dashed line represents the upper limit of the normal range. The results are plotted logarithmically. FG, foregut-derived NET excluding pancreatic NET; MG, midgut-derived NET; HG, hindgut-derived NET; unknown, unknown primary site-derived NET; MTC, medullary thyroid carcinoma

($P = 0.02$): they were low in MTC patients, higher in patients with midgut and foregut GEP NET and highest in patients with phaeochromocytoma. NSE had the highest sensitivity in patients with lung NET and phaeochromocytomas. No significant difference was found in mean NSE levels between the various groups of patients.

### *Specificity*

NSE levels were elevated in 14/53 (26%) and CgA levels in 17/53 (32%) patients with non-NET tumours (Figures 2 and 3). NSE and CgA had a specificity of 73% and 68%. Cut-off values of 19 μg $l^{-1}$ for NSE and 160 μg $l^{-1}$ for CgA were associated with a specificity of 95%, which led to a sensitivity of 19% and 38% for NSE and CgA respectively. Essential hypertension was found in 7/36 (19%) control patients with normal CgA levels compared with 2/17 (12%) patients with elevated CgA levels. In control patients, CgA was elevated in six digestive, five ovary, four breast, one lung, and one testis primary tumour, with extensive disease in 11 of them. One colon, one stomach and one breast tumour had CgA levels exceeding 200 μg $l^{-1}$. Immunostaining performed in tumours from eight control patients (three breast, two ovary, one lung, one stomach, one testis), with CgA levels ranging from 100 to 200 μg $l^{-1}$, was slightly positive, with NSE antibodies in only three and negative in all for both CgA and synaptophysin antibodies.

### Factors associated with elevated NSE and CgA levels

Elevated CgA levels were found to be significantly associated with two independent parameters, namely other secretions ($P = 0.0001$) and tumour burden ($P = 0.001$). Raised CgA serum levels were found in 66/90 (73%) patients with other secretions and in 10/38 (26%) patients with no other secretion. Mean CgA levels were found to be significantly higher in patients with other secretions (1759 ± 3119 μg $l^{-1}$) than in patients with no other secretion (249 ± 157 μg $l^{-1}$) ($P = 0.02$). When the type of secretions were analysed, only biogenic amine ($P = 0.0002$) and peptidic ($P = 0.005$) secretions were found to be significantly associated with raised CgA levels. When eutopic secretions of each type of NET was analysed, only 5-HIAA levels were found to be significantly associated with raised CgA levels ($P = 0.0001$); catecholamine secretion in phaeochromocytoma patients ($P = 0.08$) and CT secretion in MTC patients (NS) were not found to be significantly associated with raised CgA levels. Also, 68/101 (67%) patients with extensive disease and 8/27 (30%) patients with limited disease had elevated plasma CgA levels. When both parameters were taken into account in patients with limited disease, CgA had a sensitivity of 41% (7/17) when other secretions were present, compared with 10% (1/10) in patients without any other known secretion. Concerning patients with extensive disease, CgA had a sensitivity of 81% (59/73) when other secretions were present, compared with 36% (10/28) in patients without evidence of another secretion.

Raised NSE levels were significantly associated with poor tumour differentiation: they were found in 12/19 (63%) patients with poorly differentiated or intermediate cell GEP NET, and in 23/71 (32%) patients with well-differentiated GEP NET ($P = 0.01$). The other parameters studied were not significantly associated with an elevated CgA or NSE level.

### NSE and CgA in the follow-up of NET patients (Figure 1)

In all six patients in whom a follow-up study was performed, at least one other secretion was known and measured concomitantly. None of these six patients were treated with somatostatin analogues during the observation period. Five of them had progressive disease and were studied until a few months before death. CgA levels correlated with the tumour burden and the evolution of other secretions in four out of six patients, as illustrated in Figure 1. However, in one patient, despite the progression of the metastatic process, a dramatic decrease was demonstrated in CgA and also αGP levels; in another patient, the evolution of CgA and αGP was quite the opposite, with CgA correlated with the evolution of tumour burden. NSE levels correlated with the evolution of the tumour burden in three of the six patients. In the three other patients, NSE exhibited fluctuating levels (Figure 1) and was within the normal range, even during disease progression in two cases. NSE levels correlated with the evolution of other secretions in only one of the six patients.

© Cancer Research Campaign 1998

After hepatic chemoembolization, a dramatic increase was demonstrated in NSE and CgA levels remained unchanged.

### DISCUSSION

CgA proved to be a more sensitive general marker of NET than NSE with equivalent specificity. CgA and NSE had a 59% and 38% sensitivity respectively. Our results for both markers are in a lower range than that of other reported series (Cunningham et al, 1992; Nobels et al, 1997). This may be related to the size of the patient population studied and also to its characteristics: a high percentage of patients had extensive disease (80%), a high percentage had previously been treated (89%), a relatively high proportion had no known secretions (30%) and patients with small-cell lung carcinoma or neuroblastoma, in whom NSE is known to be a sensitive marker, were excluded from the study. It is noteworthy that previous treatments, including somatostatin analogue therapy, did not influence the levels of both markers. CgA levels were determined using a novel two-site IRMA, for the first time in a large series of patients. The discrepancies between our findings and those of previous studies may primarily be due to the characteristics of our assay, compared with those of conventional radioimmunoassays (RIAs) used in most studies. CgA is highly affected by C-terminal proteolysis. Conventional RIAs are based on polyclonal antibodies partly directed to the C-terminal portion, and may detect both the entire CgA molecule and peptidic products. Our assay, which allows detection of molecular forms of CgA, is based on recognition of the median domain, which is less subject to proteolysis (Degorce et al, 1996). These assays may, therefore, measure different molecular forms of CgA, but also peptides derived from CgA proteolysis. In all groups of patients with NET, CgA had a higher sensitivity than NSE. As previously reported, very high levels of CgA were found in patients with lung and pancreatic NET and in those with phaeochromocytoma.

NSE and CgA had a comparable specificity of 73% and 68% respectively. NSE has already been reported to have a low specificity (Vinores et al, 1984; Schmechel, 1985; Kaiser et al, 1989; Body et al, 1992; Nobels et al, 1997). Like other authors in initial studies (O'Connor and Bernsteir 1984: Eriksson et al, 1989), we chose the upper threshold of the normal range of CgA to attain a high level of specificity. Further studies of patients with normal and benign disease will be necessary to obtain a more accurate definition of the normal range of CgA values with this new assay. With a cut-off value fixed at 160 μg $l^{-1}$, CgA attained a specificity of 95% but sensitivity then fell to 38%. The control group has a major influence on specificity results. The elevated CgA values found in this group cannot be attributed to undetected mixed tumours in the eight patients studied, as initially suggested, nor to essential hypertension (O'Connor et al, 1989; Hsiao et al, 1991). As patients with renal insufficiency were excluded, we postulate that elevated CgA levels in the control group may have been related to the effect of stress frequently experienced by this particular population of patients with non-endocrine cancers (Cryer et al, 1991; Deftos, 1991). The influence of stress on CgA levels should then be evaluated in future studies.

Factors associated with elevated NSE and CgA levels in patients with NET other than small-cell lung carcinoma, have seldom been studied. In our study, raised NSE levels were significantly associated with poorly differentiated tumours. Previous studies claimed that NSE may reflect cell necrosis (Prinz et al, 1982; Bork et al, 1988; Kaiser et al, 1989; Schürmann et al, 1990; Cunningham et al, 1992) rather than the tumour burden, and a large population of necrotic cells is frequently encountered in poorly differentiated NET. Our findings are not in support of NSE as a direct marker of tumour burden. In contrast, CgA was found to reflect both tumour burden and tumour secretion, but independently. Elevated CgA levels may, therefore, either be related to extensive or functioning disease. As the mean CgA level differed only when the secretory activity of NET was taken into account and not the tumour burden, this result further suggests that the secretory activity of NET may be the key element influencing CgA results. Twenty-eight per cent of patients had no other secretions but elevated CgA levels and they mainly had extensive disease. This suggests that the clinical interest of CgA measurement in patients with limited disease and no detected secretion could be limited. The question as to whether increased CgA levels in patients with no other known secretion reflects undetected hormonal secretions or other mechanisms of CgA secretion, remains unresolved.

As suggested by studies on physiological mechanisms of secretions, only peptide- and amine vesicle-mediated exocytotic secretions were associated with raised CgA levels, whereas glycoprotein secretions were not (Kelly, 1985; Handwerger et al, 1987). Finally, our study demonstrated that the secretion of these two general markers may reflect different pathophysiological processes, as further suggested by the evolution of NSE and CgA levels after hepatic chemoembolization, which induced an isolated rise in NSE levels, reflecting cell lysis.

Follow-up studies of six NET patients provided further insights: CgA levels were found to correlate with tumour burden in five out of six patients. A discrepant evolution was found between the tumour burden and CgA levels in one patient, which could be related to an end stage loss of tumour differentiation. In contrast, NSE demonstrated fluctuating levels in half of the patients, precluding its use as a marker of tumour burden during follow-up.

We suggest that CgA measurement should be routinely performed in NET patients, without highly conserved eutopic secretion, such as in foregut-derived NET including non-functioning pancreatic NET. In all other NET including MTC, phaeochromocytoma, functioning pancreatic and midgut-derived NET, data derived from CgA measurement or eutopic secretions (CT, catecholamine, pancreatic peptide, 5-HIAA) should be evaluated in future studies.

In conclusion, CgA was found to be more sensitive than NSE, was found to correlate with tumour burden and demonstrated a better correlation with tumour burden evolution. We suggest that CgA ought to be the only general marker routinely screened in NET patients.

### ACKNOWLEDGEMENTS

We are indebted to Ingrid Kuchenthal, Sylvie David and Catherine Martin for secretarial assistance, to Christine Machavoine and the nurses of the Nuclear Medicine Department for technical assistance, and to Lorna Saint Ange for editing. We thank F Degorce for helpful discussion in the preparation of the manuscript. This work was supported by PHRC 1995.

### REFERENCES

Body JJ, Paesmans M, Sculier JP, Dabouis G, Bureau G, Libert P, Berchier P, Raymakers N and Klastersky J (1992) Monoclonal immunoradiometric assay and polyclonal radioimmunoassay compared for measuring neuron-specific enolase in patients with lung cancer. *Clin Chem* **38**: 748–751

© Cancer Research Campaign 1998

Bork E. Hansen M. Urdal P. Paus E. Holst JJ. Schifter S. Fenger M and Engbaek F (1988) Early detection of response in small-cell bronchogenic carcinoma by changes in serum concentrations of creatine kinase. neuron specific enolase. calcitonin. ACTH. serotonin and gastrin releasing peptide. *Eur J Cancer Clin Oncol* **24**: 1033–1038

Carney DN. Marangos PJ. Ihde DC. Bunn PA. Cohen MH. Minna JD and Gazdar AF (1982) Serum neuron-specific enolase: a marker for disease extent and response to therapy of small-cell lung cancer. *Lancet* **1**: 583–585

Cryer PE. Wortsman J. Shah SD. Nowak RM and Deftos LJ (1991) Plasma chromogranin A as a marker of sympathochromaffin activity in humans. *Am J Physiol* **260**: 243–246

Cunningham RT. Johnston CF. Irvine GB and Buchanan KD (1992) Serum neurone-specific enolase levels in patients with neuroendocrine and carcinoid tumours. *Clin Chim Acta* **212**: 123–131

Deftos LJ (1991) Chromogranin A: its role in endocrine function and as an endocrine and neuroendocrine tumour marker. *Endocrine Rev* **12**: 181–187

Degorce F. Jacquemart L. Baus MH. Bellanger L. Vidaud C and Segin P (1996) Selection of monoclonal antibodies for the measurement of chromogranin A by sandwich assay. *Clin Chem* **42**: S264

Eriksson B. Arnberg H. Öberg K. Hellman U. Lundqvist G. Wernstedt C and Wilander E (1988) Chromogranins – new sensitive markers for neuroendocrine tumours. *Ann Oncol* **7**: 453–463

Eriksson B. Öberg K and Skogseid B (1989) Neuroendocrine pancreatic tumours: clinical findings in a prospective study of 84 patients. *Acta Oncol* **28**: 373–377

Grouzman E. Gicquel C. Plouin PF. Schlumberger M. Comoy E and Bohuon C (1990) Neuropeptide Y and neuron-specific-enolase levels in benign and malignant pheochromocytomas. *Cancer* **66**: 1833–1835

Handwerger S. Wilson SP. Tyrey L and Conn PM (1987) Biochemical evidence that human placental lactogen and human chorionic gonadotropin are not stored in cytoplasmic secretion granules. *Biol Reprod* **37**: 28–31

Hsiao RJ. Parmer RJ. Takiyyuddin MA and O'Connor DT (1991) Chromogranin A storage and secretion: sensitivity and specificity for the diagnosis of pheochromocytoma. *Medicine* **70**: 33–45

Jonhson PWM. Joel SP. Love S. Butcher M. Pandian MR. Squires L. Wrigley PFM and Slevin ML (1993) Tumour markers for prediction of survival and monitoring of remission in small cell lung cancer. *Br J Cancer* **67**: 760–766

Kaiser E. Kuzmits R. Pregnant P. Burghuber O and Worofka W (1989) Clinical biochemistry of neuron specific enolase. *Clin Chim Acta* **183**: 13–32

Kelly RB (1985) Pathways of protein secretion in eukaryotes. *Science* **230**: 25–32

Nobels FR. Kwekkeboom DJ. Coopmans W. Schoenmakers HH. Lindemans J. De Herder W. Krenning EP. Bouillon R and Lamberts SWJ (1997) Chromogranin A as serum marker for neuroendocrine neoplasia: comparison with neuron-specific enolase and the α-subunit of glycoprotein hormones. *J Clin Endocrinol Metab* **82**: 2622–2628

O'Connor DT and Bernstein KN (1984) Radioimmunoassay of chromogranin A in plasma as a measure of exocytotic sympathoadrenal activity in normal subjects and patients with pheochromocytoma. *N Engl J Med* **311**: 764–770

O'Connor DT and Deftos LJ (1986) Secretion of chromogranin A by peptide-producing endocrine neoplasms. *N Engl J Med* **314**: 1145–1151

O'Connor DT. Pandian MR. Carlton E. Cervenka JH and Hsiao RJ (1989) Rapid radioimmunoassay of circulating chromogranin A: in vitro stability. exploration of the neuroendocrine character of neoplasia. and assessment of the effects of organ failure. *Clin Chem* **35**: 1631–1637

Ozturk M. Bellet D. Manil L. Hennen G. Frydman R and Wands J (1987) Physiological studies of human chorionic gonadotrophin (hCG). αhCg and βhCg as measured by specific monoclonal immunoradiometric assays. *Endocrinology* **120**: 549–558

Prinz RA. Bermes EW. Kimmel JR and Marangos PJ (1983) Serum markers for pancreatic islet cell and intestinal carcinoid tumours: a comparison of neuron-specific enolase β-human chorionic gonadotropin and pancreatic polypeptide. *Surgery* **94**: 1019–1023

Schmechel DE (1985) γ-Subunit of the glycolytic enzyme enolase: nonspecific or neuron specific? *Lab Invest* **3**: 239–242

Schürmann G. Betzler M and Buhr HJ (1990) Chromogranin A. neuron-specific enolase and synaptopysin as neuroendocrine cell markers in the diagnosis of tumours of the gastro-enteropancreatic system. *Eur J Surg Oncol* **16**: 298–303

Sobol RE. Memoli V and Deftos LJ (1989) Hormone-negative. chromogranin A-positive endocrine tumours. *N Engl J Med* **320**: 444–447

Tapia FJ. Polak JM. Barbosa AJA. Bloom SR. Marangos PJ. Dermody C and Pearse AGE (1981) Neuron-specific enolase is produced by neuroendocrine tumours. *Lancet* **1**: 808–811

Vinores SA. Bonnin JM. Rubinstein LM and Marangos PJ (1984) Immunohistochemical demonstration of neuron-specific enolase in neoplasms of the CNS and other tissues. *Arch Pathol Lab Med* **108**: 536–540

Warren WH. Gould VE. Faber LP. Kittle FC and Memoli VA (1985) Neuroendocrine neoplasms of the bronchopulmonary tract. *J Thorac Cardiovasc Surg* **89**: 819–825

© Cancer Research Campaign 1998